# Artificial Intelligence in Public Health Prevention of Legionelosis in Drinking Water Systems

**DOI:** 10.3390/ijerph110808597

**Published:** 2014-08-21

**Authors:** Peter Sinčak, Jaroslav Ondo, Daniela Kaposztasova, Maria Virčikova, Zuzana Vranayova, Jakub Sabol

**Affiliations:** 1 Department of Cybernetics and Artificial Intelligence, Faculty of Electrical Engineering and Informatics, Technical University of Kosice, Letná St. 9, Košice 04001, Slovakia; E-Mails: peter.sincak@tuke.sk (P.S.); jaroslav.ondo@tuke.sk (J.O.); maria.vircik@gmail.com (M.V.); sabolj91@gmail.com (J.S.); 2 Department of Building Services, Civil Engineering Faculty, Technical University of Kosice, Vysokoskolska St.4, Kosice 04001, Slovakia; E-Mail: vranayova@tuke.sk

**Keywords:** artificial intelligence, hot water system, intelligent buildings, neural network designed on approximate reasoning architecture, neural networks, neural subnetwork, *Legionella pneumophila*

## Abstract

Good quality water supplies and safe sanitation in urban areas are a big challenge for governments throughout the world. Providing adequate water quality is a basic requirement for our lives. The colony forming units of the bacterium *Legionella pneumophila* in potable water represent a big problem which cannot be overlooked for health protection reasons. We analysed several methods to program a virtual hot water tank with AI (artificial intelligence) tools including neuro-fuzzy systems as a precaution against legionelosis. The main goal of this paper is to present research which simulates the temperature profile in the water tank. This research presents a tool for a water management system to simulate conditions which are able to prevent legionelosis outbreaks in a water system. The challenge is to create a virtual water tank simulator including the water environment which can simulate a situation which is common in building water distribution systems. The key feature of the presented system is its adaptation to any hot water tank. While respecting the basic parameters of hot water, a water supplier and building maintainer are required to ensure the predefined quality and water temperature at each sampling site and avoid the growth of *Legionella*. The presented system is one small contribution how to overcome a situation when legionelosis could find good conditions to spread and jeopardize human lives.

## 1. Introduction

The primary goal of the paper is to present our research on a theoretical and experimental analysis of hot water distribution systems focused on reducing the microbiological risk in water systems in conditions prevailing in the Slovak Republic by using artificial intelligence principles. This research domain has developed hand-in-hand with the development of science and technology, health care and other scientific fields [1,2,3]. *Legionella*, which may cause mortality of inhabitants is very important issue around the world. Incidences in Slovakia are the lowest in the Europe. During the years 1985–2013 there were 88 reported cases (55 male, 33 females) in our country [4], eight of them mortal. Extensive study of this problem in Slovakia can be found in [5]. *Legionella pneumophila* occurring in water distribution systems can grow and multiply, therefore it is important to reduce and monitor them [2,3,6,7,8]. The in-building distribution system is an important part of the water network that has a big influence on the water quality at the consumer’s tap. The conditions that encourage the bacterial grow are: bacteria presence, a temperature range between 20–50 °C, slow moving or stagnant water, adequate food source, formation of aerosols, and the presence of people. We must break this chain to ensure that the system will be safe. Information technology support is a way how to overcome this threat and use a simulation and virtualization approach to simulate an environment which could lower the risks or bacterial contamination in water systems. The challenge is to create a community access system which will be able to simulate a virtual water distribution system in the building, including a water tank temperature profile which is the most common place for bacterial occurrence.

To present the current state of art and importance of the issue we describe only some examples from the latest recorded cases around the world [9]. Dangerous levels of *Legionella* bacteria, which cause potentially fatal Legionnaire’s Disease, were discovered in Sydenham, UK, and the Health Protection Agency investigated a hospice because two of its patients and one of its employees contracted the disease in January 2013. An Indianapolis hospital restricted patients’ showers and baths with provision of bottled water for drinking after two patients contracted Legionnaires’ disease in January 2014. The Michigan Department of Community Health reported a higher than normal number of Legionnaires’ cases occurred in the metro Detroit area in June 2013. In the same year the town of Warstein in Germany survived one of the worst legionella outbreaks in its history, when more than 160 cases were detected [6,8,10,11]. An outbreak of Legionnaires’ disease affecting 65 people in the cities of Ulm and Neu-Ulm, in South-west Germany, was detected in January 2010 [12,13]. These are only some of the latest recorded cases from around the world [6].

## 2. Artificial Intelligence in Intelligent Buildings

We use the term “intelligence” in the current world in connection with many things like cars, games, systems, *etc.*, and buildings. We can find a lot of definitions in the literature [14], depending on the domain. There is no formal definition of intelligent buildings in Slovakia. Intelligent buildings are utilizing an Automated Building Management System (ABMS), which are connected by various technical equipment, sensors, controllers, communication protocols. The infrastructure of the building has to be pre-programmed, but a program is provided learning ability from environment and intelligent building users [15]. The aim of “artificial intelligence” (AI) is to develop methods and algorithms that, after their implementation in the system, allow the system to solve problems in the way they would be dealt with by a human [16]. Thus, the system can be described as artificially intelligent, if it successfully solves a set of tasks in similar ways to human solutions [6,7]. In addition, every intelligent system must be able to store knowledge, apply knowledge to solve the problem and also be able to acquire new skills during the experiments, so it must be able to learn [16]. Artificial Intelligence is based on learning ability and so called robustness, so the main advantage of the Artificial Intelligent solution is ability to learn, adapt and incrementally not-forget situations for the future. We thus think that Artificial Intelligence approaches have no alternatives if we are considering autonomous systems for human benefit. The main contribution of Artificial Intelligence is to reduce the need for human care and involvement in routine control and adaptation processes. Humans are shifted to a role of observer and less and less human intervention is expected in Artificial Intelligent systems. All aspects and technologies of our lives are heading towards “smart planet” as described in an IBM Company policy [17]. Neural networks are the most suitable since they are function on approximations and are based on data, so data is the source of knowledge and can easily react to different situations which are hidden in the data. The neuro fuzzy system NARA provides a good tool to partially understand data in the form of IF-Then rules encoded into the neural network. This is the reason why neuro-fuzzy network solutions are preferred for this research. 

The learning ability of an intelligent building can be implemented using various technologies, including soft computing methods. Learning ability can be used also for safety issues, including the prevention of Legionnaires’ disease caused by hot water systems [13]. In order to control the water temperature and the risk of *Legionella* colonization in the system, we propose an approach based on neural networks which have as input the flow and temperature to the water tank and the output is a temperature profile of the water tank and selected nodes of the water system. The collected data are trained in the neural network to provide an approximation tool to predict a temperature profile for the input data. The goal is to obtain a virtual water tank simulation based upon various inputs of flow and temperature of the incoming water.

Hodkiewicz [3] used a Bayesian Belief Network (BBN) model to assess the impact of changes in maintenance tactics on risks and associated costs for managing a cooling water system. According to her, the Bayesian approach offers an intuitive way to develop models based on the combination of statistical data and domain experts.

Armero *et al.* [18] implemented a probabilistic expert system capable of predicting the risk of Legionella in real time from remote information relating to the quality of the water in evaporative installations. The inference engine of the expert system is constructed through Bayesian networks. Bayesian reasoning and Markov Chain Monte Carlo algorithms are applied in order to study the relevant unknown quantities involved in the parametric learning and propagation of evidence phases. A modelling framework using fuzzy-based methods was implemented by Sadiq *et al*. [19]. Similarly, Iglesias and Pelansky [20] mentioned among the enhancements of their research on a profile-based control for central domestic hot water distribution the use of fuzzy-decision algorithms. They claim that soft computing methods present good features to deal with the uncertainties and partial truths that are inherent to the prediction of human behaviors and they expect that fuzzy controllers will better manage profile data to optimally activate the recirculation and adjust setpoint temperatures.

Our proposed model is based on the fusion of artificial neural networks and fuzzy inference systems in order to combine the advantages of the both soft-computing techniques. As stated, e.g., by Nayak *et al.* [21] and Lohani *et al.* [22] the advantage of neuro-fuzzy methods is that they do not require the model structure to be known *a priori*.

### 2.1. Research Goals and Methods-Legionella Contamination in Kosice (Slovakia)

Representative data from water samples were collected from private homes, hospitals and boiler houses of Kosice. The selection was made on the basis of the water distribution systems inside the town and buildings and heater types in each area and in connection to our previous research where physical modeling of circulation in tanks and transport in pipelines was presented [13] and the experimental storage was simulated with the software Fluent 6.3 to identify the risk parts of the boiler.

Data with bacteria were recorded in eight samples of potable water hot (PWH). In potable water cold (PWC) waters for human consumption, a volume of *Legionella* was detected ranging from sporadic colonies of 20 Colony Forming Units-CFU/100 mL up to massive colonization with a quantity of 6700 CFU/100 mL per sample. There volume of *Legionella* detected in PWH water for human consumption ranged from sporadic colonies of 200 CFU/100 mL up to massive colonization on the order of 14,600 CFU/100 mL per sample 

We reacted promptly due to positive bacteria identification in residential areaa. Thermal disinfection was the most reliable solution in these cases. However, the non-adjustment of the system and areas not reached by the thermal disinfection lead to fast spreading of bacteria in the whole system [23,24]. In our experiment, by using Water Quality Management methods, especially Risk Analysis, the water heater tank was verified as a primary source of *Legionella* [13]. To prevent the growth of *Legionella* bacteria, the water temperature must be in such a range that the bacteria will not grow or have minimum growth (>60 °C). During normal use, the hot water temperature in the installation should be capable of reaching minimum 55 °C at any point [2,23,25,26,27,28]. This process can be computationally simulated with using Artificial Intelligence tools, so we decided to create a virtual hot water tank to predict the temperature profiles. A computational model of the water tank should help perform virtual experiments and set up the proper conditions to prevent the existence of *Legionella* in the system. The plan is to perform long term testing of the proposed system to achieve feedback from the users and provide some potential improvements of the system. We have addressed four specific goals:

1. To analyse and control the existing centralized hot water system in the experimental building for experimental data collection,
2. To create a software framework for simulation of the temperature profile in the system,
3. To create a virtual hot water tank as part of the simulated system with Artificial Intelligence tools, namely-neuro-fuzzy systems, as a precaution against legionelosis,
4. To propose the extension of the hot water tank simulator by adding a possibility to connect any other hot water tank, retrain neural networks and simulate water temperature profiles in the connected hot water system.

### 2.2. Hot Water Distribution System in the Building of the Faculty of Civil Engineering, Kosice *(*Slovakia*)*

The building of the Faculty of Civil Engineering, Technical University of Kosice (FCE), is supplied with hot water from a centralized hot water distribution system. The distribution substation is located in the basement of the FCE building, from which not only the FCE building is supplied with hot water but also several surrounding buildings. In terms of technology, hot water system in the FCE building is considered to be controlled. It is a water management system involving a heat exchanger, in which water is heated, and a hot water tank. This system is equipped with two hot water tanks connected in parallel, each with a capacity of 2000 litres.

Cold water enters the hot water system at a flow rate of M1 and temperature t1. Cold water flow is measured by a traditional billing water meter. The entering cold water mixes with the heated water which has not yet been consumed and is delivered back to reheating by a circulation pipe in the mixing node. This water has temperature t2 and a flow rate M2. Cold water mixed with hot water produces water at a flow rate of M3 and temperature t3. Flow rates of M2 and M3 should be the same as it is a closed system. The mixed water enters the settling tank where it is cleaned from sludge and from there it is moved to the heat exchanger. The exchanger heats the water to the required temperature. Heating is based on a circulatory flow of the hot water system. 

**Figure 1:**
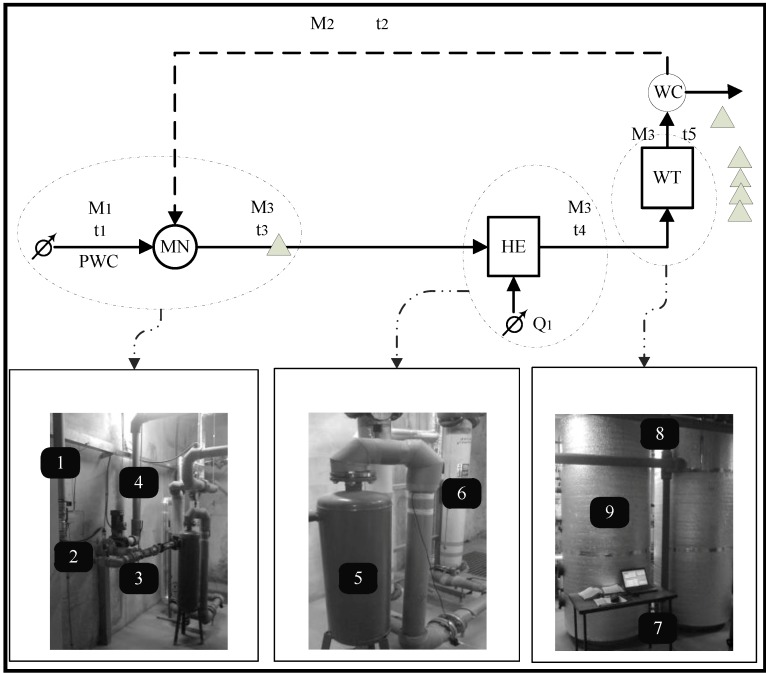
A Schematic diagram of the hot water exchanger with the locations where the simulation of water temperature was proposed and with the photographs of specific parts 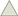 indicates a computer prediction temperature.

*Legend*: PWC—portable cold water; MN—mixing node; HE—heat exchanger, water heating; WT—hot water tank; WC—water consumption; M1—quantity of cold water; M2—circulatory flow; M3—water supply to the storage tank; t1, t2, t3, t4, t5—water temperature in different parts of the system; Q1—the amount of heat delivered; 1—cold water inlet; 2—billing water meter; 3—mixing node; 4—circulation pipe; 5—settling tank; 6—heat exchanger; 7—inlet of storage tanks; 8—outlet of storage tanks; 9—with two hot water tanks connected in parallel.

The hot water storage tank keeps the heated water ready for distribution to customers and is designed to produce a minimum heat loss. The role of the tank is to deliver a sufficient supply of hot water to cover the times of increased hot water demand by customers in order to avoid supply disruptions at times of increased hot water consumption. From the hot water storage tank, the water is distributed to customers in the surrounding buildings. The water that is not consumed is returned back into the circulation pipe of hot water system for reheating. This clearly shows that the system is closed. Finally, we should note that the flow of M1 is consistent with the consumption of hot water. That implies that the amount of hot water leaving the system is equal to the amount of cold water entering the system for heating (see Figure 1).

### 2.3. Simulation of the Water Tank System

The simulation of water temperature profile in the hot water storage tank from a mathematical point of view involves identification of the unknown function relating the amount of hot water used and the water temperature profile in the hot water storage tank. The simulation framework can provide an estimation of the water temperature profile corresponding to the given input values.

We proposed to simulate six water temperatures in various parts of the water tank system. The first one is the temperature t3, composed of the water temperature of mixed cold water and the water supplied by circulation pipes. Next simulated water temperatures are tz1, tz2, tz3 a tz4—temperatures in four layers of the hot water storage tank. The last simulated temperature is t5, which is the water temperature at the outlet of the hot water tank.

For each temperature simulation in the hot water system and the hot water tank, we suggest using a separate neural network. In particular, in simulations of water temperatures in the hot water tank, we considered the possibility that the output of the neural network simulating the water temperature in the lower layer of the hot water tank may contribute to the input of the neural network simulating water temperature in a higher layer of the hot water tank. We have designed an experimental procedure and the entire procedure consists of four basic steps:

1. Collecting the necessary data for experimental work;
2. Modelling and processing the data, preparation of training and testing sets needed for training the neural networks;
3. Programming a computer program to simulate the water temperature profile in the tank;
4. Proposal and implementation of the computer program extension. An extension allows to connect and adapt computer program and simulate water temperature profile in any hot water tank.

## 3. Collection and Processing of Data Necessary for Experimental Work

The collection of the necessary data for experimental work was carried out in the heat exchange substation located in the basement of the FCE building from 25 October 2011 to 15 November 2011. The data collection lasted continuously for 21 days. During the data collection, the working days and public holidays alternated, which enabled us to obtain data recording water temperature profiles in the hot water tank under various different water consumption conditions. Nine thermometers were installed in various parts of the hot water distribution system. They were located at the points of the hot water system where we planned to simulate the water temperature, or in the places where we assumed that these records could form the input of the designed neural network. Each temperature probe recorded current temperatures in minute intervals. The capacity of each meter was 10,400 records, which was sufficient to record approximately a week–long measurement. In addition to automated meters, we manually carried out meter readings of cold water consumption and energy consumed for heating the water two to three times a day. These data allowed us to determine the flow of water in different parts of the hot water distribution system in minute intervals.

Given that approximately 25,000 temperature readings were collected and many data from each sensor were recorded in a separate file, it was necessary to model the data in a form which was suitable for further processing. Based on the data, it was found that the sensors are not fully reliable and there were occasional recording dropouts lasting several minutes, so we had to find the missing values and take them into consideration and eliminate mistakes.

The next step in processing the collected data was the recalculation of manual consumption readings in minute intervals. The recalculation of flow rates was necessary as manual reading of these values was carried out two to three times a day and the water temperature was measured in different parts of hot water system in minute intervals.

The actual recalculation was carried out in two steps—by determining the relative flow rates *m*_1_ and *m*_2_ and their approximation according to the consumption of cold water in a given time interval based on the relations between equations (1) and (2). In (2), cwc stands for cold water consumption:

*m*_1_ = *m*_2_[(*t*_3_ − *t*_2_)/(*t*_1_ − *t*_3_)] [kg/hr] (1)

*M*_2_ = *m*_2_[cwc/∑*m*_1_] = constant  [kg/hr] (2)

We acquired the *M*_2_ as the actual flow of water in the circulation pipe. This flow rate is constant. Finally, we had to determine *M*_1_ and *M*_3_ flows according to the relations between equations (3) and (4):

*M*_1_ = *M*_2_[(*t*_3_ − *t*_2_)/(*t*_1_ − *t*_3_)] [kg/hr] (3)

M_1_ is the actual flow of cold water, *M*_2_ is the actual flow in the circulation pipe, t_3_ is the temperature of cold water mixed with the water delivered by the circulation pipe, t_2_ is the temperature of the water delivered by the circulation pipe, and t1 is the temperature of cold water entering the system:

*M*_3_ = *M*_1_ + *M*_2_ [kg/hr] (4)

*M*_3_ is the actual flow of cold water mixed by the circulation pipe in the hot water system.

### 3.1. Neuro-Fuzzy Systems for Temperature Simulation

**Figure 2:**
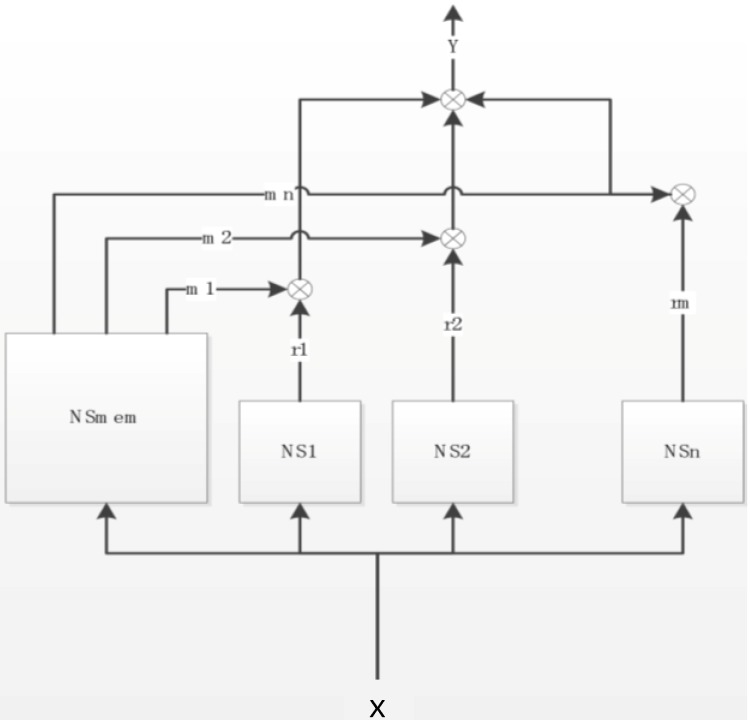
The conceptual scheme of a NARA neural network with neural sub-networks.

The NARA architecture belongs to a class of hybrid neuro-fuzzy systems and was used in this project. The acronym NARA comes from the English name “Neural Network Designed on Approximate Reasoning Architecture”, suggesting that the neural network is designed to approximate the decision-making architecture. This neural network (NN) is structured and constructed in accordance with the structure of IF-THEN fuzzy rules [29]. Takagi *et* *al.* [30] stated that since the proposed model can implement explicit knowledge in NN using a rule structure, it is reasonable that the proposed structured neural network has higher performance than an ordinary neural network from the viewpoint of the relation between NN capacity and information amount in a task. The most important feature of a NARA neural network is a complex which consists of two types of neural networks (sub-networks)—One Kohonen memory type neural subnetwork (NSmem) and others are neural feed forward sub-neural networks (NS1, …, NSn) (see Figure 2). The input data analysis made by the Kohonen neural network gives a user better knowledge about the data structure and approximation function in general. The NARA model can be simply described. An unknown sample, which comes to the input of the NARA model, is classified into any of found groups, called clusters which are identified in the Kohonen neural network (NSmem). Depending on where the sample is passed, one or more feed forward parallel neural networks (NS1, …, NSn), which are experts on the cluster identified in Kohonen network, will have an essential influence on the output of the NARA neural network. So basically each of the essential clusters in the NSmem network has a relevant NS neural network. Then an “Y” output (see Figure 2) is the weighed output by NSmem based on the outputs of the NS1, … NSn neural subnetworks. Based on the entering input “X”, the NARA architecture determines the final output which represents the simulated temperature pertaining to a given input [29]. Controlling water temperature in the hot water distribution system is one of the possibilities how to use artificial intelligence to protect the human health and support the building to be intelligent.

**Figure 3:**
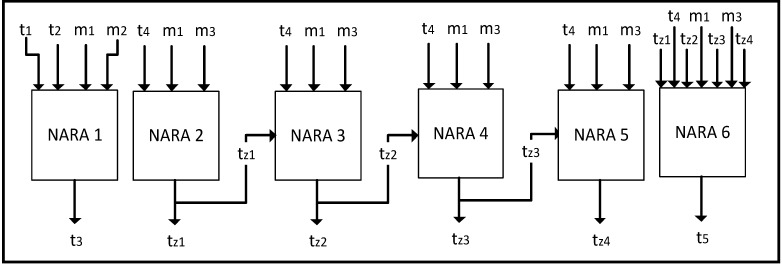
Proposed NARA models. Inputs are temperatures and flow rates. They are in the upper part of the figure (*t*_1_, *t*_2_, …, *t*_z3_, *t*_z4_). Outputs are temperatures of water in proposed points of the hot system.

The work proposes to use a NARA model to simulate each temperature in the hot water tank. We have used six different NARA neuro-fuzzy systems to be able to predict six different temperatures in the water tank which represent a temperature profile of the water tank (see Figure 3). Computational complexity of the problem is not so hard since NARA is a fast learning neuro-fuzzy system if we function like data and so we are able to simulate a functional relation between the input and output of the system.

## 4. The Implementation of a Computer Program to Simulate the Water Temperature Profile in the Hot Water Tank

The implementation of a program to simulate the water temperature profile in the hot water tank was the penultimate step. The very first program was implemented by using the programming language C# as a Windows application. Despite the possibilities using any other programming languages (e.g., C, C++, Java *etc.*), we chose the C# language due to its easy programming, quick implementation of the user interface, rich support and advanced programming environment. On the one hand, the programs developed in C programming language could execute operations much quicker, contrary to C# language, but on the other hand C# is natively supported by Visual Studio, which contains many features and allows creating graphical user interfaces in a simple manner. The other reason for using the C# language is effectivity of programming and the planned future implementation into the Cloud Computing environment AZURE which is easy to implement if using the C# programming language. C# also provides a hardware independent implementation when the .NET framework is working in either the Windows or Linux environments.

The program is used to determine the water temperature profile in the hot water tank corresponding to the given input. The mentioned program is an implementation of NARA architectures for each simulated value. The program is easy to use. After entering the required input values, the program sequentially determines simulated temperature values in the hot water system and hot water tank. For each simulated temperature, the program uses the NARA model which is trained for simulating this temperature. On the basis of NARA model, the water temperature is specified in the corresponding entry (see Figure 3).

As mentioned in Section 3, we suggested the simulation of six temperatures in the hot water system in the FCE building. Six different NARA models were implemented for the simulation of these temperatures. Two models were used to simulate the temperature values in different parts of the hot water system. Specifically, we simulated the water temperature of cold water mixed with already heated water from the circulation pipe for reheating. The second NARA model was designed to simulate the water temperature at the outlet from the hot water tank. The remaining four NARA models were designed to simulate the water temperature in four layers of the hot water tank.

**Figure 4:**
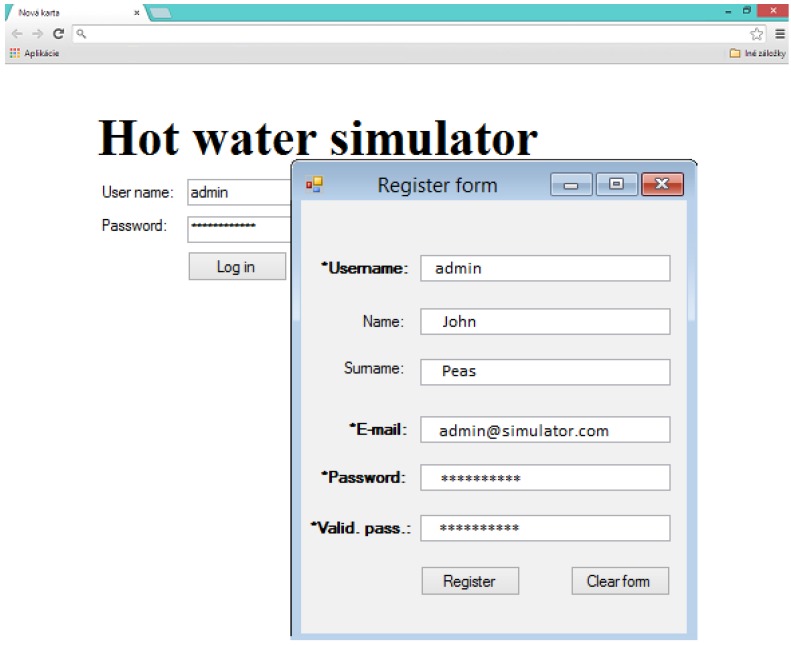
Web interface for the designed application.

Hot water systems such as the one shown on Figure 1, and also the one which was implemented in our simulator, are used very frequently in a wide spectrum of public and private buildings. This implies that the described hot water tank simulator is suitable for generalization. The differences between specific hot water systems are caused by different capacities of hot water tanks, the number of customers which use hot water from one hot water tank, and also by the intensity of the hot water usage. All these factors determine the hot water system characteristics and the behaviour of a water temperature profile in the hot water tank. The hot water tank simulator must adapt according to specific attributes of the hot water system. Therefore, we proposed a windows form application transformation into the web application. The transformed computer application was extended by adding new functionalities, like the possibility to create user accounts; login, training of user-defined neural networks, setting the hot water system parameters, saving the results, *etc* (see Figure 4).

**Figure 5:**
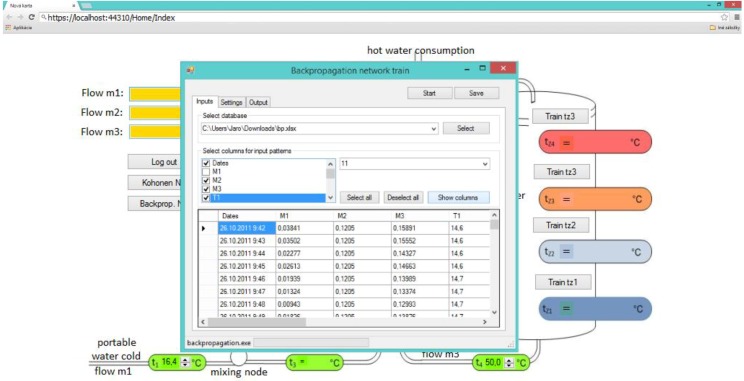
The user interface of the Web based Virtual Water tank.

A web application (see Figure 5) was implemented in C# programming language as a web form user interface and also by using VisualWebGui. VisualWebGui is a strong multi-platform tool which allows developing web, cloud or smart phone applications. It provides a high level web server protocol. The applications created by this tool are secured HTML5 web pages. The choice of this technology is because it provides a good and effective environment for web- and cloud-ready programing. The user has to collect necessary training data from a real hot water system which the user wants to simulate. Training and testing sets have to be created from the collected data. When this step is finished, the user has to train every NARA architecture for the prediction of temperatures in different parts of hot water systems. Before the neural network training, the user must set the right parameters of neural networks, like learning parameter, find and set correct topology, *etc.* It is necessary to train every neural network several times until the neural network provides the smallest possible error. Once every NARA model is trained, the neural network parameters are saved and the simulator is ready to work. The user can use the test set to verify the simulation results. The adaptation process looks user unfriendly, but the web application provides a very intuitive user interface which guides the user through the entire process.

## 5. Results and Discussion

The results show that test simulation of hot water system placed in the basement of Faculty of Civil Engineering is quite accurate. The precision of the simulation depends on the quality of collected data and training quality of every NARA model (see Table 1) and we can state that accuracy was rather precise and in 97 percent of cases we have achieved a temperature profile. After the implementation of extensions of the very first program, we can adapt the simulator to similar hot water systems and we can predict the temperature profile in these systems.

In general, the simulation of water temperature in the lowest layer of the hot water tank is the least accurate. Provided we have good quality data and precisely trained neural networks, we can simulate the water temperature profile in the tank to within ±0.5 °C from the real temperature of the water in the tank for more than 97% of all possible input values. The worst obtained result during testing the proposed model was ±1.5 °C of the real temperature.

**Table 1:** Results of prediction using NARA neuro-fuzzy system.

Node	ID of Found Cluster	No. of Badly Classified Samples	Total Samples in the Cluster	Total Error [%]	Total Accuracy [%]	Error Tolerance [°C]
*t*_3_	1	20	5207	0.38%	99.62%	±0.1
	2	29	14,536	0.20%	99.80%	
	3	5	8923	0.06%	99.94%	
	4	1	35	2.86%	97.14%	
*t*_z1_	1	12	5160	0.23%	99.77%	±0.5
	2	16	10,690	0.15%	99.85%	
	3	48	11,203	0.43%	99.57%	
	4	7	1447	0.48%	99.52%	
*t*_z2_	1	738	24,732	2.98%	97.02%	±0.5
	2	53	2542	2.08%	97.92%	
	3	23	1026	2.24%	97.76%	
	4	7	200	3.50%	96.50%	
*t*_z3_	1	32	15,139	0.21%	99.79%	±0.5
	2	68	11,829	0.57%	99.43%	
	3	18	1532	1.17%	99.83%	
*t*_z4_	1	31	11,624	0.27%	99.73%	±0.5
	2	19	14,193	0.13%	99.87%	
	3	4	1283	0.31%	99.69%	
*t*_5_	1	325	12,330	2.64%	97.36%	±0.3
	2	396	13,286	2.98%	97.02%	
	3	28	1484	1.89%	98.11%	

There are some limitations in the predictions. The main obstacle of NARA is that if some event can occur which was not in the history the neuro-fuzzy system must be retrained for that new situation. The new situation is defined in a case where the hot water tank is continually providing a water and temperature profile of the water tank which is determined by water consumption and the water users’ preferences. The maintainer of the simulation model has to train NARA system for these situations. Among the planned future modifications of the system are an increased autonomy of the neuro-fuzzy training. Also the possibility to find an optimal neural network topology and training parameters automatically by using intelligent methods, like evolutional algorithms, can be added. The good news that this is incremental process and the system is learning from each new situation and the maintainer of the system should have less and less service inference. That means that a simulation system has ability of incremental learning and the simulations become more mature and robust.

## 6. Conclusions and Future Research

Artificial Intelligence tools are starting to be widely used in a number of civil engineering applications [31,32,33,34,35,36]. We have focused our interest on a particular part of hydroinformatics. The prevention of dangerous bacteria in water distribution systems is an important topic. A wide range of factors support the growth of *Legionella* and other microorganisms in a water distribution system. Because of the health significance of these organisms, it is necessary to pay particular attention to the issues of design and implementation of preventive medical and technical measures and control systems. This paper describes the use of NARA neuro-fuzzy system to simulate a virtual water tank and its temperature profile with sufficient accuracy. This tool should be useful in prevention of *Legionella* bacteria flowing in hot water systems, regardless of whether a centralized or decentralized hot water system is used in buildings. We can watch how the temperature varies in different parts of the water tank and determine in which part of the system the risk of colonization by *Legionella* increases due to the presence of a consistent water temperature which is ideal for colonization. The future of this research is in AZURE cloud computing implementation and further work on a more universal solution of the simulation so it could be used in more general environments. The NARA neuro-fuzzy system is being well able based on theory and experiments to handle large amounts of data.
